# Carbon nanoparticles adversely affect CFTR expression and toxicologically relevant pathways

**DOI:** 10.1038/s41598-022-18098-8

**Published:** 2022-08-22

**Authors:** Torben Stermann, Thach Nguyen, Burkhard Stahlmecke, Ana Maria Todea, Selina Woeste, Inken Hacheney, Jean Krutmann, Klaus Unfried, Roel P. F. Schins, Andrea Rossi

**Affiliations:** 1 IUF – Leibniz-Research Institute for Environmental Medicine, Auf’m Hennekamp 50, 40225 Duesseldorf, Germany; 2 IUTA – Institut für Energie- und Umwelttechnik e.V., Duisburg, Germany; 3 Medical Faculty, Heinrich Heine University, Düsseldorf, Germany

**Keywords:** Cell biology, Environmental sciences, Risk factors

## Abstract

Cystic fibrosis is an autosomal recessive disorder caused by mutations in the cystic fibrosis transmembrane conductance regulator (CFTR) that can lead to terminal respiratory failure. Ultrafine carbonaceous particles, which are ubiquitous in ambient urban and indoor air, are increasingly considered as major contributors to the global health burden of air pollution. However, their effects on the expression of *CFTR* and associated genes in lung epithelial cells have not yet been investigated. We therefore evaluated the effects of carbon nanoparticles (CNP), generated by spark-ablation, on the human bronchial epithelial cell line 16HBE14o− at air–liquid interface (ALI) culture conditions. The ALI-cultured cells exhibited epithelial barrier integrity and increased *CFTR* expression. Following a 4-h exposure to CNP, the cells exhibited a decreased barrier integrity, as well as decreased expression of CFTR transcript and protein levels. Furthermore, transcriptomic analysis revealed that the CNP-exposed cells showed signs of oxidative stress, apoptosis and DNA damage. In conclusion, this study describes spark-ablated carbon nanoparticles in a realistic exposure of aerosols to decrease CFTR expression accompanied by transcriptomic signs of oxidative stress, apoptosis and DNA damage.

## Introduction

Cystic fibrosis transmembrane conductance regulator (CFTR) is an important cAMP-regulated chloride channel in the cell membrane of human airway cells. Next to the contribution to mucus hydration and obstruction^1^, CFTR has several other functions and is necessary for proper lung physiology^2^. *CFTR* mutations can lead to the development of cystic fibrosis (CF). Interestingly, CF patients display phenotypic variability, even among patients carrying the same mutation^3^. CF phenotypes can be influenced by environmental factors and exacerbated under air pollution conditions^4,5^.

In urban areas, particulate air pollution predominantly consists of ultrafine combustion-derived carbon particles^6^. Depending on the emitting source, these may be associated with contaminating toxicants like transition metals and organic compounds, including polycyclic aromatic hydrocarbons, that have been shown to contribute to their toxic effects^7–9^. Carbon nanoparticles (CNP) are considered a good model for the carbonaceous core of environmental ultrafine particles. Studies investigating the effects of pure CNP demonstrate that these particles are able to induce adverse health effects mostly based on inflammatory responses in rodents and humans^10–12^. The cellular and molecular mechanisms which cause pro-inflammatory and other adverse health effects are closely linked to the reactive oxygen generating properties of CNP and associated generation of oxidative stress in lung cells and tissues^8,13^. Besides DNA damaging effects, intracellular reactive oxygen species can trigger signaling cascades which lead to the production and release of pro-inflammatory cytokines and chemokines^14–16^. While consensus has developed about the importance of carbonaceous nanoparticles in air pollution induced lung diseases like chronic obstructive pulmonary disease (COPD), their specific effect on CFTR and the pathology of CF to the best of our knowledge has not yet been fully elucidated. Decreased CFTR expression has direct consequences in CF patients but it has also been proposed to play a role in non-CF in patients suffering from COPD^2^. CFTR expression in human bronchial epithelial cells has been shown to be reduced with exposure to cigarette smoke^17–19^. In vitro studies with pro-oxidant substances suggest the hypothesis that the reduction of CFTR protein levels may be caused by oxidative stress^20^. The effects of carbonaceous nanoparticles exposure to 16HBE14o^−^ at air–liquid interface (ALI) culture including transcriptomic landscape, CFTR and cystic fibrosis modifier gene expression have not been fully studied yet.

In order to address this question, we evaluated the effects of pure CNP in the human bronchial epithelial cell line 16HBE14o-, upon their controlled exposure at air–liquid interface (ALI) culture conditions. These cells were selected because they retain several features of normal differentiated bronchial epithelial cells, including a normal karyotype, the the ability to form an epithelial barrier^21^ and can be cultured in ALI conditions^22,23^. Moreover, 16HBE14o^−^ cells express CFTR at the apical cell surface^24^, making this cell line an ideal model for CF-related research^25^ even though they do not recapitulate all the features of primary epithelial cells.

Various studies have already been carried out with 16HBE14o^−^ cells to explore mechanisms of toxicity of ambient particulate matter (PM) and nanoparticles in the lung^26–28^. Notably, we previously showed that these cells, similar to alveolar epithelial cells, respond to CNP exposure with pro-inflammatory signaling events which were also observed in vivo in exposed rodents^29^. Diesel exhaust particles showed effects on the barrier function of 16HBE14o- cells including reduced expression of tricellulin (*TRIC*), decreased transepithelial electrical resistance (TEER), increased fluorescein-isothiocyanate (FITC)-Dextran permeability^30^ and altered occludin (*OCLN*) expression^31^. A transcriptomic analysis of 16HBE14o- cells exposed to airborne PM2.5 revealed upregulation of genes involved in xenobiotic metabolism and inflammatory pathways, which was predominantly attributed to the absorbed substances, like PAHs^32^.

As with most in vitro investigations with nanoparticles, the aforementioned studies were carried out in cells under submerged conditions where particles were administered in suspension. However, interactions of nanoparticles with the suspension medium change the properties of the particles and therefore also the effects on cells^33^. To achieve more realistic particle exposure conditions, approaches have been developed that combine ALI exposures of lung cell models with aerosol generation devices^27,34–37^.

Therefore, in the present study we exposed the 16HBE14o- cells in an automated exposure station^35,38,39^ at ALI conditions, using the controlled generation of CNP aerosols by spark-ablation. Here we report that CNP exposure leads to decrease CFTR expression accompanied by transcriptomic signs of oxidative stress, apoptosis and DNA damage.

## Material and Methods

### Cell culture

16HBE14o- cells (RRID:CVCL_0112) were kindly provided by Dr. D. Gruenert (University of California, San Francisco, CA) and cultured in α Minimum Essential Medium (SIGMA, St. Louis, MO, USA) supplemented with 10% fetal bovine serum (Thermo Fisher, Waltham, MA USA), 1% L-glutamine (Thermo Fisher) and 1% Penicillin/Streptomycin (Thermo Fisher) at 37 °C with 5% CO_2_. Culture flasks were coated with a sterile filtered extracellular matrix (ECM) solution comprising human fibronectin (SIGMA, 10 µg/mL), bovine serum albumin (SIGMA, 100 µg/mL) and collagen (SIGMA, 30 µg/mL). Cells were maintained in ECM-coated culture flasks. For experiments, 6 × 10^5^ cells were seeded in non-coated Transwell inserts (Corning, PET, 24 mm, 0.4 µm pore size) with culture medium and cultured for 4 days under submerged conditions. At day 4 after seeding apical medium in the inserts was removed and the cells were cultured under ALI conditions for 14 days to characterize cell properties. The CNP exposure experiments were carried out at day 5 of ALI culture. Basolateral medium was replaced every two days.

### Transepithelial electrical resistance (TEER) measurements

The development of an epithelial barrier was assessed via TEER measurements, recorded with ERS-2 Volt-Ohm-Meter with STX01 chopstick electrodes (Millipore, Burlington, MA, USA) on each day of submerged culture and on distinct days of ALI culture. Medium was replaced in both Transwell compartments and cells were incubated at 37 °C for 25 min and subsequently kept at room temperature for 2 min before measuring TEER. Values were recorded for each of the three cavities of one insert and the mean was calculated. The values of a blank insert without cells were subtracted as the background value.

### FITC-Dextran permeability assay and immunostaining

Cellular barrier integrity was additionally assessed using 4 kDa FITC-Dextran (SIGMA) permeability as described previously^30^. Medium was removed from both compartments, cells and the wells washed with PBS. Basolateral wells were filled with 1.5 mL DMEM high glucose without phenol red (Thermo Fisher) and 500 µL of 5 mg/mL FITC-Dextran (dissolved in the same DMEM) were added to the apical compartment. Cells were incubated for 2.5 h at 37 °C and sample fluorescence of the basolateral medium was measured in triplicates in a black 96 well plate in a Tecan Spark plate reader (Tecan, Männedorf, Switzerland) with excitation at 490 nm and emission at 520 nm against a FITC-Dextran standard curve.

16HBE14o- cells ZO-1 (sc33725, Santa Cruz, CA, USA) staining under ALI conditions was performed according to the manufacturer's protocol using as secondary antibody a Goat IgG anti-Rat IgG (H + L)-Alexa Fluor 488 (Thermo Fisher, A-11006) and the Hoechst 33,342 Solution (Thermo Fisher, 62,249). Samples were imaged with a Leica DMi8 Thunder Imager (Leica, Microsystem, Wetzlar, Germany) and Thunder imaging processing system.

### Generation and characterization of the CNP

**Figure 1:**
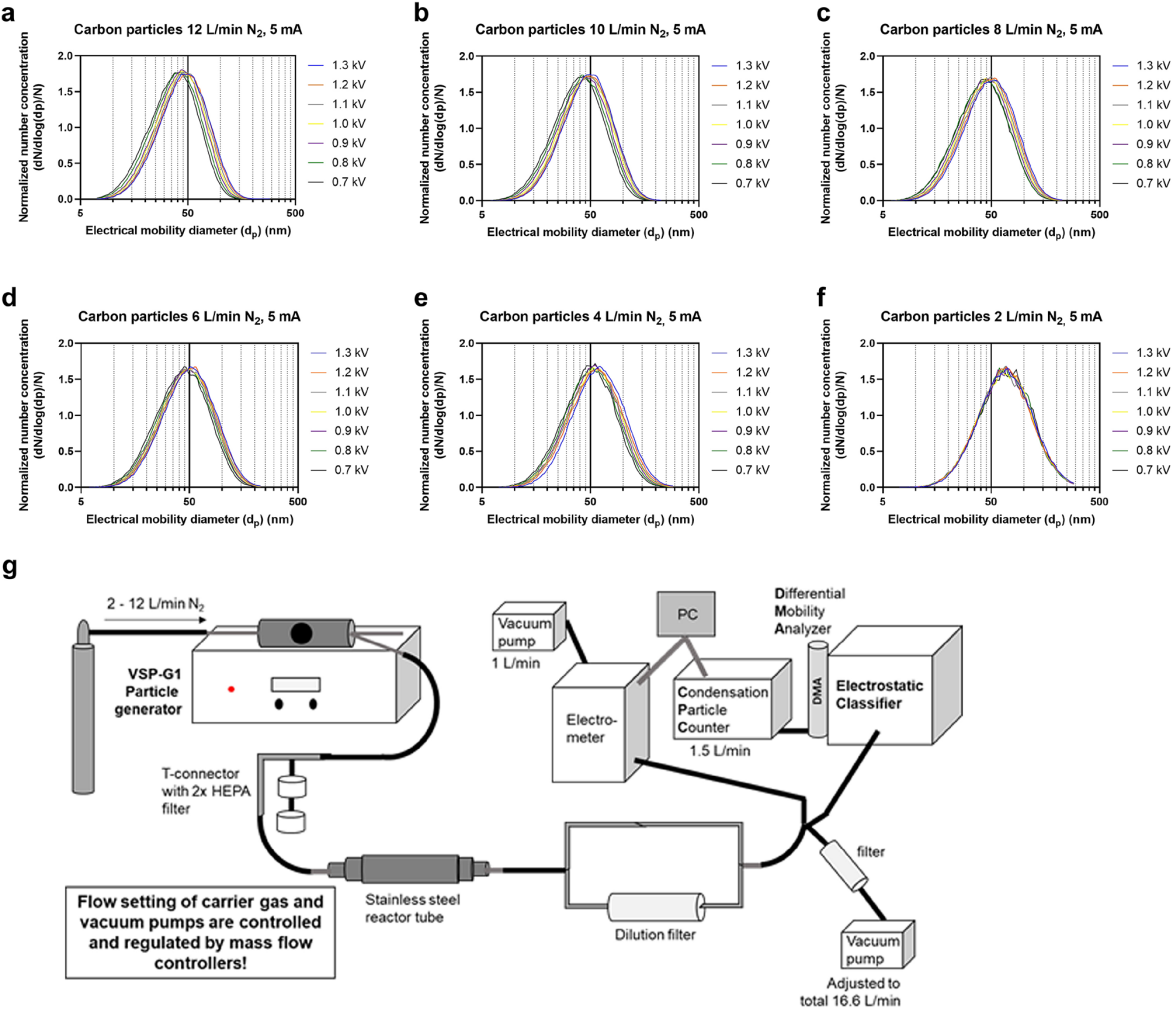
Physical, morphological and chemical characterization of CNPs generated by spark ablation. (**a–f**) Normalized particle number size distributions for different aerosol generator settings; carrier gas flow (2–12 L/min), gap voltage 0.7–1.3 kV. (**g**) Schematic of the setup used for the initial physical characterization of spark-ablated carbon nanoparticles. VSP-G1 nanoparticle generator was supplied with nitrogen carrier gas at 2–12 L/min flow. Generated particles were diluted with room air at the T-connector with two HEPA filters and guided through a tube system to be subsequently measured with a Scanning Mobility Particle Sizer consisting of an electrostatic classifier with a Differential Mobility Analyzer (DMA) and a Condensation Particle Counter (CPC), and an Electrometer. Appropriate flow parameters in the tubes and devices were set by vacuum pumps and regulated with mass flow controllers. Graphic was generated with the graphic tool from Microsoft Power Point.

The CNP were generated by the method of spark ablation using the VSP-G1 particle generator (VSParticle, Delft, Netherlands) equipped with graphite electrodes and supplied with nitrogen as carrier gas. Prior to the cell exposure studies, different CNP generation conditions were compared for the VSP-G1 generator to evaluate effects on particle size distribution characteristics and number concentrations, as described in Fig. 1. Number particle size distribution and concentration were measured with a Scanning Mobility Particle Sizer (SMPS, model 3936, TSI Inc., Shoreview, MN, USA) equipped with a Differential Mobility Analyzer (DMA, model 3081, TSI Inc.) and a Condensation Particle Counter (CPC, model 3776, TSI Inc.). The DMA was operated at 10.5 or 15 L/min sheath flow and 1.5 L/min aerosol flow rate. The particle induced charge was measured with an electrometer (model 3086B, TSI Inc.). For each spark generator settings three 180 s long SMPS scans were recorded and the mean of these measurements were calculated.

The mean number of charges per particle (n_p_) was calculated according to Eq. (1)

1 $${n}_{p}=\frac{I}{e\times N\times {q}_{e}}$$

I = electrical current (A), e = elementary unit of charge, 1.602 × 10^–19^ Coulombs, N = particle number concentration (particles/cm^3^), q_e_ = flow rate (cm^3^/sec).

### Exposure of cells to CNP

16HBE14o- cells exposures, at the air–liquid-interface, were carried out using the VITROCELL® Automated Exposure Station (VITROCELL SYSTEMS GMBH, Waldkirch, Germany)^35,38^. Based on the CNP characterization experiments, a flow rate of 8 L/min nitrogen carrier gas at 1.0 kV gap voltage with 5 mA was chosen for the ALI exposures. Accordingly, the generated test atmosphere was mixed with filtered room air by a HEPA-filter to a flow of 16.6 L/min and guided into the 37 °C heated and 85% humidified aerosol reactor of the Automated Exposure Station, resulting in a calculated final concentration of oxygen of approximately 10.9%. From the aerosol reactor the particles were guided with 100 mL/min by isokinetic sampling to the exposure modules with the cells. Three inserts with cells were exposed to CNP for 4 h, while three additional inserts were exposed to humidified and pressurized clean air as control samples (CAC, 21% O_2_). In one additional exposure module the CNP were sampled for characterization on transmission electron microscopy (TEM) grids (nickel-based grids with continuous carbon film, 400 mesh size, Plano GmbH, Germany), immobilized in a TEM grid holder (Vitrocell)^40^. During exposure, cells were supplied with 25 mM 4-(2-hydroxyethyl)-1-piperazineethanesulfonic acid (HEPES)-supplemented culture medium from the basolateral side. The deposited mass of the CNP generated with these parameters (i.e. mean diameter of 44 nm, particle number concentration of 6.36 × 10^6^/cm^3^) was estimated to be approximately 230 ng of CNP after 4-h exposure (Supplement 1). For this calculation we considered 1.5% of the applied particles as deposited on the cells^35^. Considering the surface area of the insert this corresponds to 50 ng/cm^2^.

### Electron microscopy/Energy dispersive x-ray spectroscopy (EDS) analysis of CNP

For the morphological evaluation of the CNP the obtained TEM-grids were analyzed by means of Scanning Electron Microscopy (EM) applying a Tescan CLARA RISE (Tescan GmbH, Dortmund, Germany) high-resolution scanning electron microscope at an acceleration voltage of 15 kV and a current of 100 pA. Typically, a zoom series with increasing magnification was obtained at a representative position of the grid. The elemental composition of the particles was analyzed by applying energy dispersive x-ray analysis (EDAX Octane Elect detector, AMETEK GmbH, Wiesbaden, Germany), confirming the purity of the generated CNP.

### RNA isolation and qPCR analysis

After measuring TEER and FITC-Dextran permeability subsequent to exposure, cells were washed with PBS twice and homogenized in TRIzol for RNA isolation using the column-based Direct-zol RNA preparation kit (Zymo Research, Freiburg, Germany). Five hundred ng of total RNA were used for cDNA synthesis with the High-capacity RNA-to-cDNA Kit (Thermo Fisher). Gene expression was measured with KAPA SybrFast qPCR mastermix (SIGMA) on a QuantStudio 3 device (Thermo Fisher) with oligonucleotides for different target genes (Supplement 2). *PGK1* was used as reference gene and expression was calculated using the ΔΔCt-method^41^.

### Protein isolation and western blot

Total protein was extracted with 1 × RIPA lysis buffer (Cell Signaling Technologies CST, Danvers, MA, USA) containing protease and phosphatase inhibitors (Roche, Basel, Switzerland). The cleared lysate was measured for protein concentration with Pierce BCA assay (Thermo Fisher). Thirty µg total protein (4X LDS buffer) were denatured at 90 °C for 5 min and separated on a 4–12% Bis–Tris gel under denaturing conditions with MOPS buffer and transferred on a PVDF membrane via semi-dry Turbo Blotting for 30 min. Two different membranes were blocked with 5% BSA/TBS-T and incubated respectively with primary antibodies against CFTR (Santa Cruz Biotechnology Cat# sc-376683, RRID:AB_11151574, 1:500) and beta-actin (Cell Signaling Technology Cat# 3700, RRID:AB_2242334, 1:1000) overnight at 4 °C in 5% BSA/TBS-T. Secondary-HRP conjugated anti-mouse antibody (Cell Signaling Technology Cat# 7076, RRID:AB_330924, 1:2000) was incubated at room temperature for 60 min and bands were detected with Western Bright Chemiluminescence Substrate (Advansta Inc., San Jose, CA, USA) on a LICOR device. Band intensities were analyzed with the Image Studio Lite software (Image Studio Lite, RRID:SCR_013715). In order to test CFTR antibody specificity, 16HBE14o- cells were transfected with *CFTR* siRNA (Ambion Silencer, s534180) using Lipefectamin 2000 (Thermo) or Nucleofection (Amaxa, Lonza) according to the manufacturer's protocol and protein expression. 3 days after transfection CFTR expression was analyzed using WB analysis (data not shown). Original WB figure (Supplement 8) and metrics can be downloaded at https://tinyurl.com/2mtjuc9w.

### Library preparation and sample loading for long-read nanopore RNA-Sequencing (RNA-Seq)

RNAseq experiments were performed as previously described^42^. Briefly, quality of isolated RNA was determined using the High Sensitivity RNA Screen Tape System (Agilent Technologies, Santa Clara, CA, USA). All samples showed a RIN value > 7.7. Library preparation, including reverse transcription and multiplexing of the samples was performed with the PCR cDNA Barcoding Kit (Oxford Nanopore Technologies, Oxford, United Kingdom) using 50 ng total RNA. Quantity of amplified cDNA was measured with the Qubit 4 Fluorometer (Invitrogen, Carlsbad, CA, USA) and the fragment size was examined using the Agilent D1000 ScreenTape assay (Agilent Technologies). The SpotON flow cell (R9.4.1, FLO-MIN106D) was prepared with the Flow Cell Priming Kit (EXP FLP002, Oxford Nanopore Technologies) and equal amounts of barcoded cDNA were loaded to a total of ~ 100 fmol. Sequencing was carried out with a MinION device (MN33710) using the MinKNOW software (v.21.02.1) over a period of 72 h.

### Basecaller and quality control

Oxford Nanopore (ONT) raw fast5 reads were converted into fastq files by the guppy GPU basecaller (version 5.0.11 + 2b6dbffa5) with super-accurate model dna_r9.4.1_450bps_sup.cfg. We used a standard quality control ONT MinIONQC^43^ and a general-purpose FastQC to examine the read quality and detect abnormalities in base quality score, GC content, sequence duplication levels, adapter content, and read length.

### Sequence alignment, gene count, and differential expression analysis

Sequence reads were aligned to the Genome Reference Consortium GRCh38 using Minimap2^44^ and Samtools^45^ to create BAM files with optimized parameters for Nanopore. Feature Counts/Rsubread^46^ was used to calculate the gene count matrices, then aligned reads are mapped to reference transcripts using Ensembl 102 dataset. We also used HTSeq^47^ to ensure no abnormal mapping to genome annotation by mutual gene counts matrices from two methods. To analyze the differential expression we use DESeq2 and Duesselpore^48,49^ that shows the reliable capability of processing RNA-Seq in a small number of replicas.

### Statistical analysis and time

All exposure experiments were performed and repeated with three biological replicates and all analyses used at least two technical replicates. Exact conditions and statistical tests are depicted in the figure legends. Unless otherwise stated the graphics were generated and statistical significance was calculated using GraphPad Prism 8 software (GraphPad Prism, RRID:SCR_002798). Experimental timing was taken using an Omega Speedmaster Professional caliber 1863.

## Results

### CNP characterization under aerosol exposure conditions

CNPs were generated using different parameter combinations, *e.g.* carrier gas (N_2_) flow and gap voltage. All number particle size distributions were lognormal and in the range between 5 and 300 nm (Fig. 1a–f). Different parameter combinations of carrier gas flow and gap voltage were used to establish the desired CNP size characteristics for the ALI exposure experiments. The measurement setup (Fig. 1g) therefore represented the conditions (total flow, tube lengths and tube diameters) that exist under the exposure conditions in the Automated Exposure Station.

### Characterization of 16HBE14o- barrier integrity in ALI culture conditions

**Figure 2:**
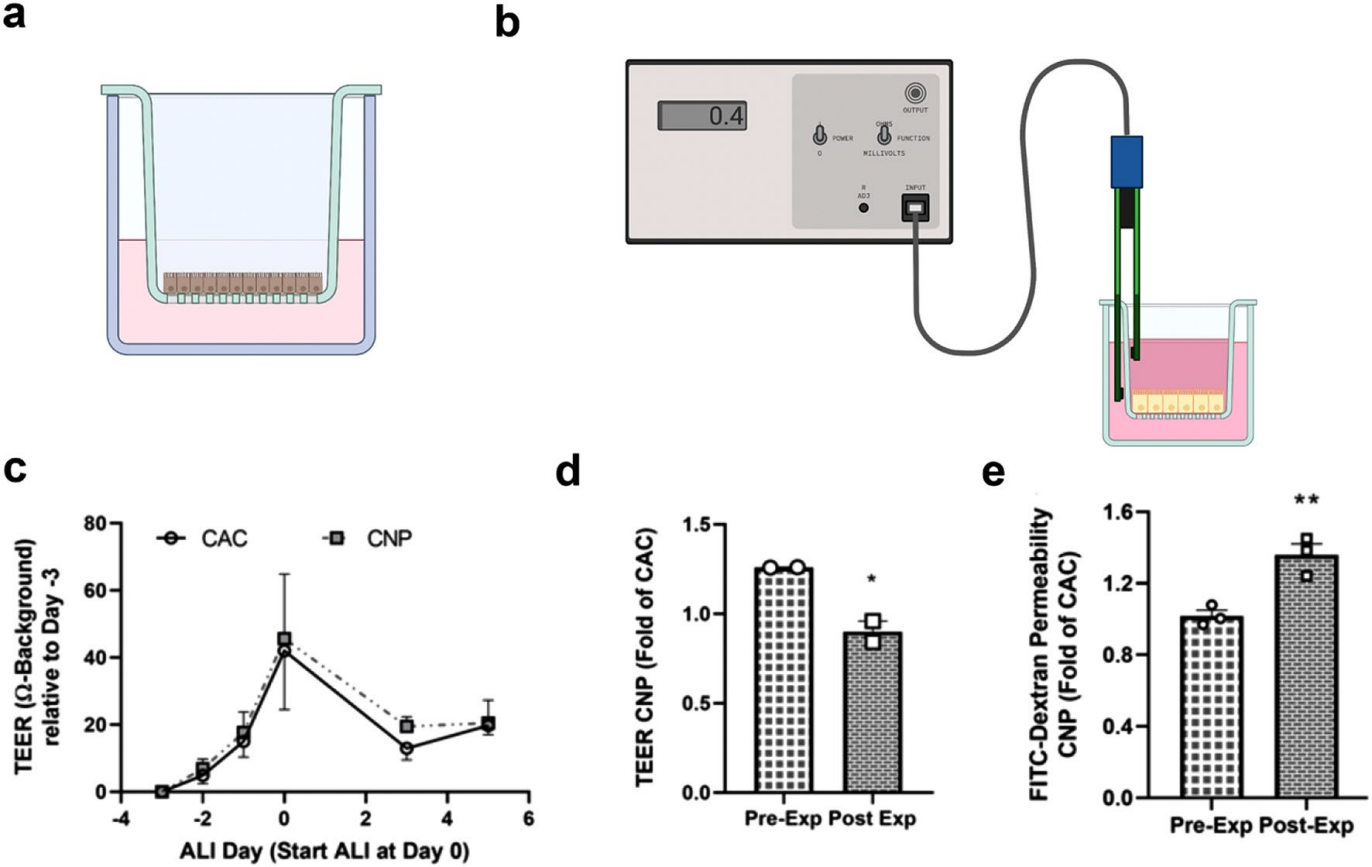
Assessment of barrier integrity in ALI-cultured 16HBE14o- cells pre- and post-exposure. (**a**) Schematic representation of ALI-cultured 16HBE14o- cells (**b**) Schematic representation of a TEER device is depicted (**c**, **d**) TEER was measured as described in the method section before and after ALI start until day 5 (pre-exposure) and expressed relative to TEER values at day -3. Data are mean ± SEM of four independent experiments with three Transwell inserts for each experiment. Each Transwell insert was measured at three cavities and the mean was recordedb TEER was measured directly before (pre-exposure) and after (post-exposure) 4-h CNP or CAC exposure and expressed relative to TEER values at day -3. Data are mean ± SEM of two or three independent experiments with three Transwell inserts for each experiment. (**e**) FITC-Dextran permeability was measured one day before (pre-exposure) and directly after (post-exposure) CNP or CAC exposure. Data are mean ± SEM of three independent experiments with three Transwell inserts for each experiment. Two-tailed, unpaired student ‘s t-test; *p < 0.05; **p < 0.01.

The barrier integrity of cells under ALI condition (Fig. 2a) was assessed by measuring TEER (Fig. 2b, Supplement 3a), FITC-Dextran permeability (Supplement 3b) and mRNA expression of markers for tight junctions, adherens junctions and *CFTR* (Supplement 3c). TEER values were similar to those observed before for 16HBE14o- cells (Supplement 3a, b)^38,39^. FITC-Dextran permeability assay was performed to quantify transepithelial permeability. Until day 3 of ALI culture, FITC-Dextran permeability was approx. 0.55 mg/mL, corresponding to approx. 72% of the cell-free permeability indicating no tight barrier between the cells. Permeability at day 6 dropped to approx. 0.25 mg/mL, corresponding to 25% of the cell-free permeability and remained around 0.3 mg/mL until day 14 of ALI culture (Supplement 3b). We investigated mRNA expression of markers for cell–cell contact and *CFTR* (Supplement 3c). *CLDN1* expression steadily increased to approx. fourfold at day 6 and approx. 11-fold at day 14, relative to day 0. Expression of the adherens junction gene *CDH1* (E-cadherin) steadily increased to approx. twofold after 14 days of ALI culture. Expression of *CFTR* also increased during ALI culture between day 6 and day 14 (Supplement 3c). Furthermore, 16HBE14o- cells appear to exhibit polarity as suggested by ZO-1 and nuclei staining (Supplement 3d). All together, these data indicated that the 16HBE14o- cells under ALI condition are able to form an appropriate morphological and functional barrier to model airways in physiological conditions and in response to insults such as toxicants and pathogens.

### Effects of CNP on barrier integrity

To evaluate the effects of CNP on the epithelial barrier integrity, we measured TEER during the culture of the cells, directly prior to and after a 4 h exposure interval. TEER development between different Transwell was not different when referred to the starting value at day −3 (Fig. 2c). TEER values were similar in both groups before CNP-exposure and it is particularly highlighted when expressing the TEER values of the CNP-exposed cells as fold of CAC which is significantly lower post-exposure compared to pre-exposure (Fig. 2d). We also measured FITC-Dextran permeability in the CNP exposed cells. Values for pre-exposure were measured one day before the exposure, whereas post-exposure was measured after TEER measurement directly after the 4 h exposure. CNP-exposed cells had a significantly higher fold of FITC-Dextran permeability post-exposure compared to pre-exposure (Fig. 2e). These data indicate a decreased barrier integrity in CNP-exposed cells.

### CFTR expression and assessment of oxidative stress

**Figure 3:**
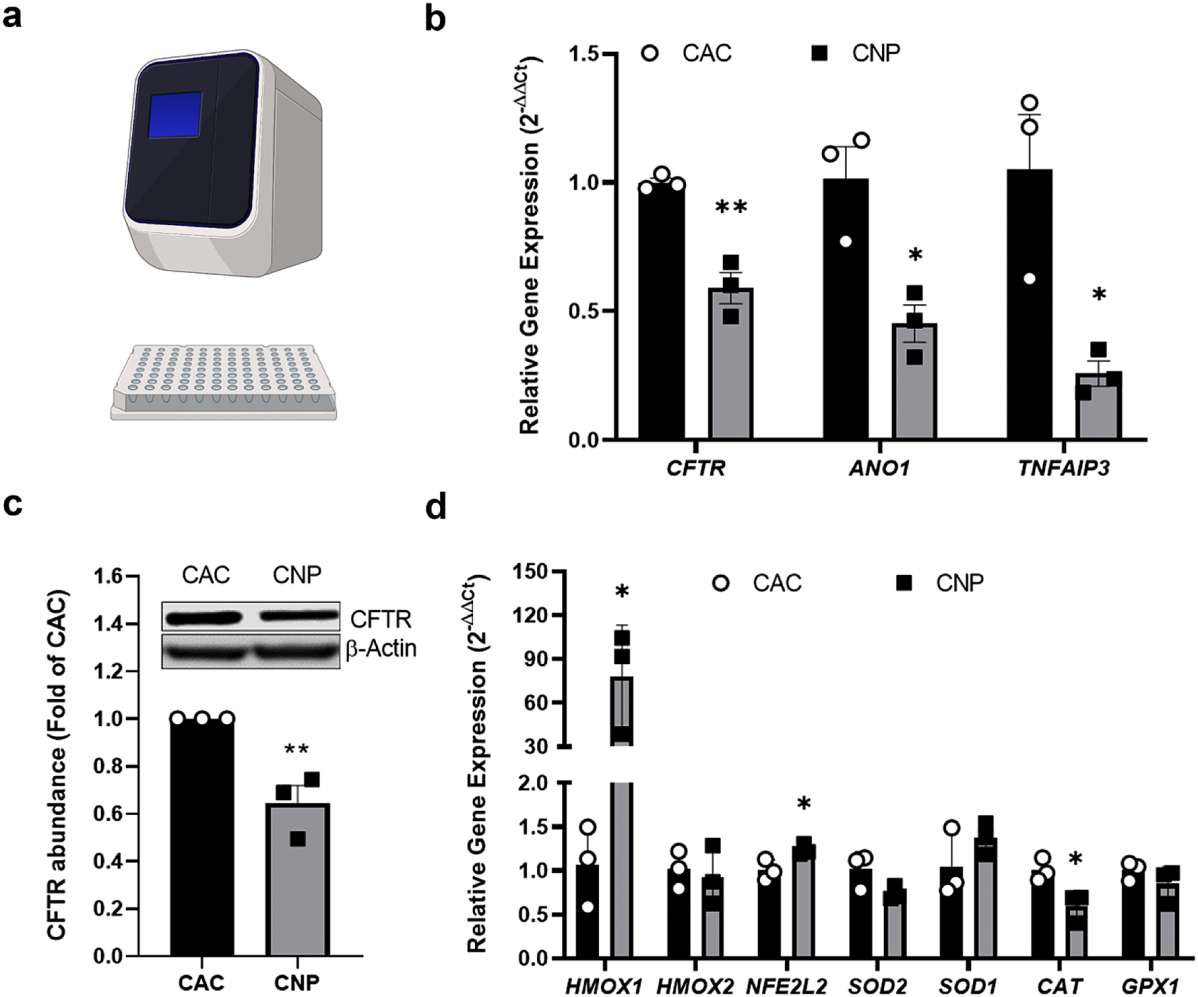
Analysis of CFTR and oxidative stress related genes expression (**a**) Schematic representation of a qPCR device and a plate used for the analysis. (**b**) ALI-cultured 16HBE14o- cells were exposed to spark-ablated CNP generated with 8 L/min nitrogen gas flow, 1.0 kV gap voltage and 5 mA at day 5 of ALI and gene expression of *CFTR*, *ANO1* and *TNFAIP3* was measured by qPCR, normalized to *PGK1* expression. Data are mean ± SEM of three independent experiments. Each experiment was measured with two technical replicates. Two-tailed, unpaired student ‘s t-test, * *p* < 0.05, ** *p* < 0.01. (**c**) From the same cells proteins were isolated and 30 µg protein were subjected to western blot analysis (cropped image, depicted are lane 4 and 5 of Fig. S9 with the full western blot image with 3 biological replicates) for CFTR and β-Actin as reference protein. Data are expressed as fold of CAC samples. Data are mean ± SEM of four independent experiments. Two-tailed, unpaired student ‘s t-test, **p < 0.01. (**d**) A panel of markers for oxidative stress was analyzed by qPCR. Data are mean ± SEM of three independent experiments. Each experiment was measured with two technical replicates. Two-tailed, unpaired student ‘s t-test, * *p* < 0.05.

In order to assess *CFTR* gene expression in CAC and CNP-exposed cells, real time qPCR experiments were performed (Fig. 3a). *CFTR* mRNA expression was significantly downregulated by around 40% in CNP-exposed cells compared to clean air (Fig. 3a). Interestingly we also found two cystic fibrosis modifier genes, *ANO1* (TMEM16A) and *TNFAIP3*, downregulated by 60% and 70% respectively, in CNP-exposed cells (Fig. 3b). Decreased expression of CFTR was also apparent on protein level (Fig. 3c), although further experiments are necessary to determine the underlying mechanism. The interaction of lung cells with CNP has been associated with the generation of reactive oxygen species leading to intracellular oxidative stress^13^. Moreover, studies of Cantin et al., indicated an association between pro-oxidants and the loss of CFTR^20^. We thus performed real time qPCR experiments targeting genes involved in pro- and antioxidative cellular responses in cells after exposure to CNP (Fig. 3d). *HMOX1* expression was drastically increased by approx. 80-fold in CNP-exposed cells. Likewise, expression of *NFE2L2* as transcription factor for the regulation of redox balance and protective antioxidant responses (including *HMOX1* induction) was significantly increased. In contrast, catalase gene expression (*CAT*) was significantly downregulated, while *SOD1, SOD2* and *GPX1* expression were unchanged. Of note, exposure to the nitrogen-air mixture alone did neither influence barrier integrity nor gene expression, indicating that the observed effects are particle related (Supplement 4).

### CFTR expression and oxidative stress markers using RNA-Seq analysis

**Figure 4:**
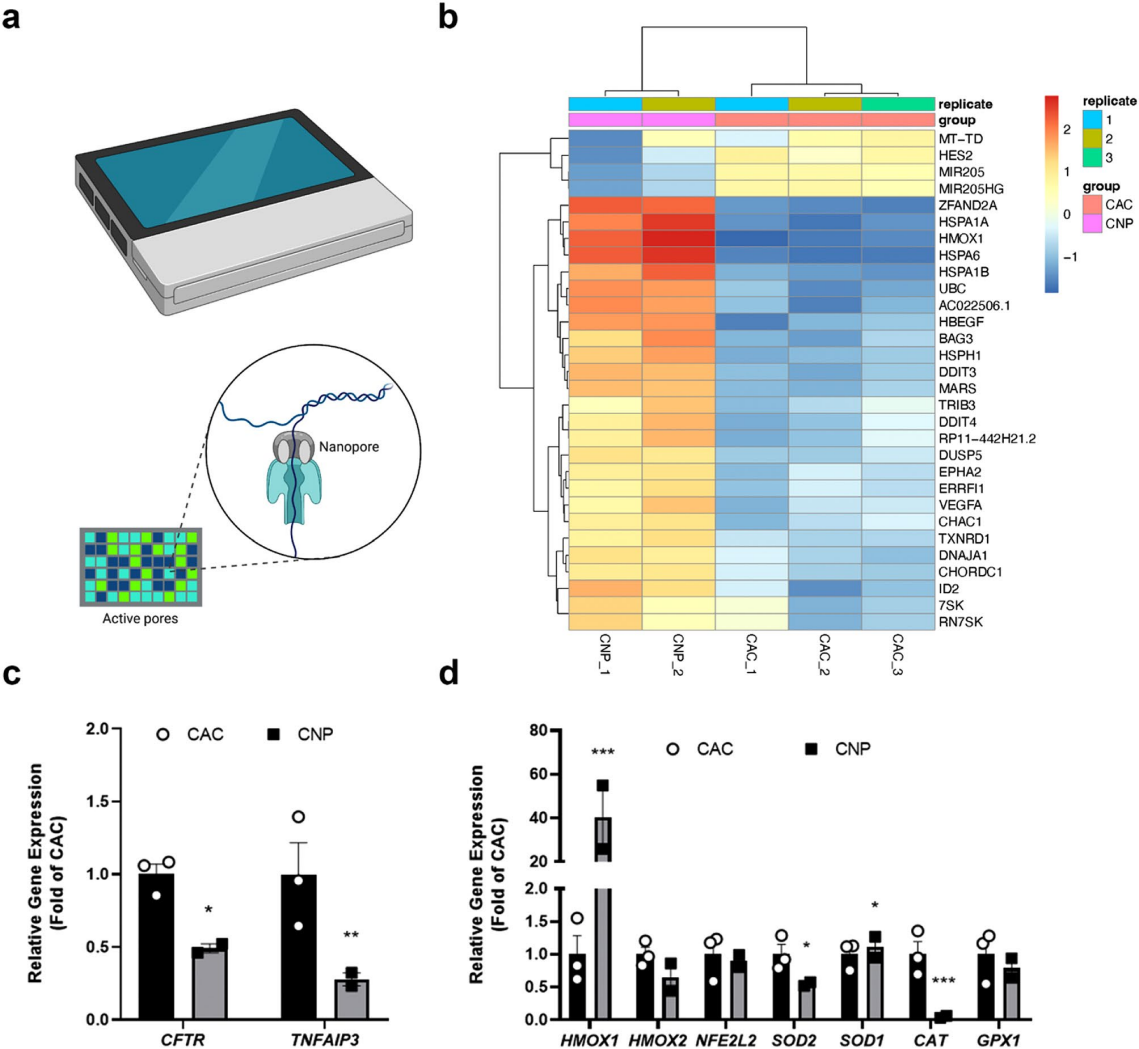
RNA-Seq analysis of 16HBE14o- cells exposed to clean air control or spark-ablated CNP. (**a**) RNA-Seq analysis was performed using a minION device (ONT, top). DNA or RNA molecules pass through a pore and cause alterations in electric signal when passing through the pore (bottom). Eventually, these signal fluctuations are further translated (basecalled) into distinct bases with special algorithms (**b**) Heat Map analysis of RNA-Seq data. Visualized are the 30 top differentially expressed genes in a heatmap plot and genes ranked by Wald test p-value, then clustered by hierarchical clustering (**c**) *CFTR* and *TNFAIP3* expression or (**d**) oxidative stress markers. Data are expressed as fold of clean air control (CAC). Data are mean ± SEM of two (CNP) or three (CAC) independent experiments. Statistical significance was calculated using the DESeq2 algorithm as described in the method section. *p < 0.05; **p < 0.01; ***p < 0.001.

Oxford Nanopore Sequencing (Fig. 4a) was used to analyze the transcriptomic landscape of 16HBE14o- cells after CNP exposure. Heat Map analysis of RNA-Seq data identified several differentially expressed genes (Fig. 4b). *CFTR* and *TNFAIP3* expression were downregulated to a comparable extent as with qPCR (Fig. 4c) while *ANO1* expression was not detected. Furthermore, the expression pattern for oxidative stress markers showed slightly different results to the qPCR measurement (Fig. 4d). In the RNA-Seq dataset, *NFE2L2* was not differentially expressed amongst different samples. In contrast, *SOD2* expression was significantly downregulated, while *SOD1* expression was significantly upregulated. *CAT* expression was clearly downregulated and hardly detectable in CNP-exposed cells.

### RNA-Seq revealed further effects in CNP-exposed cells

**Figure 5:**
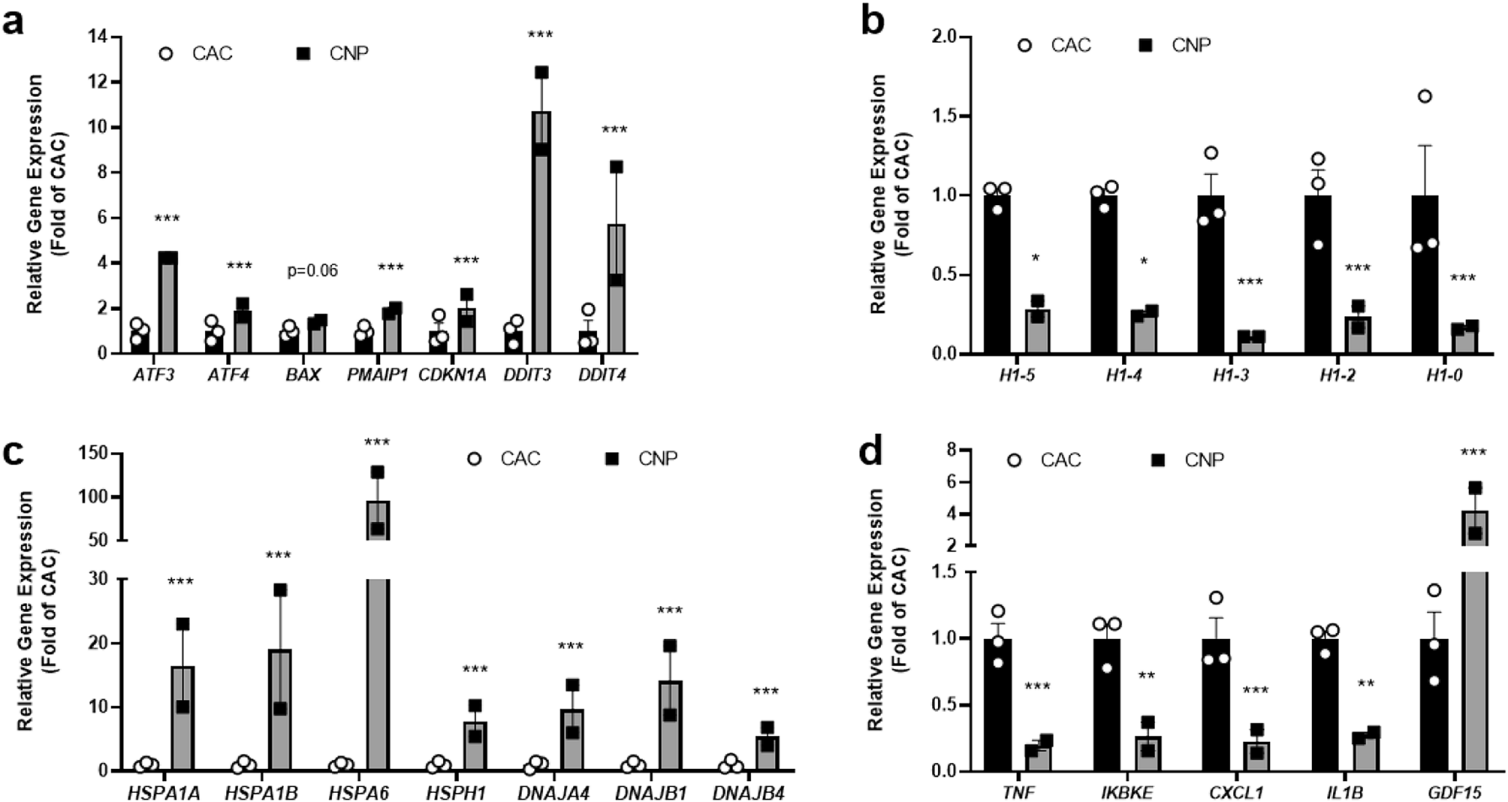
Differentially regulated genes analyzed by RNA-Seq reveal further adverse effects of CNP on 16HBE14o- cells. Isolated RNA was subjected to Long Read Nanopore Sequencing to analyze gene expression of different markers involved in apoptosis and DNA damage (**a,b**), heat shock and DNA-binding (**c**) or inflammatory processes (**d**) Data are expressed as fold of CAC. Data are mean ± SEM of two (CNP) or three (CAC) independent experiments. Statistical significance was calculated using the DESeq2 algorithm as described in the method section. *p < 0.05; **p < 0.01; ***p < 0.001.

Downregulation of *CFTR* and the induction of oxidative stress upon CNP exposure can potentially affect the expression of other genes. Thus, while performing RNA-Seq data analysis, we focused on differentially expressed genes belonging to apoptosis, DNA damage, cell stress and inflammatory pathways. Strongly increased expression of *ATF3*, *ATF4*, *PMAIP1*, *CDKN1A*, *DDIT3* and *DDIT4* was indicative of activated apoptosis pathway and DNA damage in CNP-exposed cells (Fig. 5a). This was supported by significantly decreased expression of different histone 1 subunits (Fig. 5b). Strongly increased expression of various heat shock protein genes and DNA-binding protein genes in CNP-exposed cells suggest that they are in a stress state (Fig. 5c). We also measured markers for the inflammatory pathway and show significantly differentially regulated genes (Fig. 5d). *TNF*, *IKBKE*, *CXCL1* and *IL1B* were downregulated while *GDF15* was highly upregulated in CNP-exposed cells.

## Discussion

Carbon nanoparticles account for a substantial proportion of ambient particulate matter in urban environments and are nowadays well recognized to cause adverse effects in lung cells^14,50,51^. Inhalation of particulate matter as the major component of outdoor air pollution affects human health and exacerbates symptoms in chronic airway disease patients such as in CF^52,53^. CF patients with mutated *CFTR* show highly variables phenotypes, even among patients with the same mutation^3^ suggesting that environmental factors may influence the severity of the disease. CFTR expression is fundamental for lung function, nevertheless its expression after CNP exposure in human lung cells has not been studied before. So here, we wanted to test whether CNP influence *CFTR* expression and if the environment can account for these discrepancies. Thus, we first established an ALI system with cells that have been extensively used in CF research and toxicological studies. By culturing 16HBE14o- cells at the ALI we showed a developing barrier integrity, indicated by decreased FITC-Dextran permeability. This is supported by increasing *CLDN1* gene expression during ALI culture of the cells suggesting this protein as a major contributor to that barrier. Moreover, increase in *CFTR* expression during ALI culture suggests that cell polarity develops, since CFTR incorporates predominantly into the apical cell membrane. The formation of a cell polarity was also suggested by ZO-1 immunostaining (Supplement 3d) as also previously shown by He et al.^39^.

Using the particle generator VSP-G1, we generated pure CNP with a mean diameter of approximately 44 nm and a particle number concentration of 6.36 × 10^6^/cm^3^. With these parameters, we calculated the particle mass deposited on the cells after 4 h exposure to be approx. 50 ng/cm^2^. This calculation was based on the mean particle diameter and not on the number particle size distribution. However, due to the presence of agglomerates, the effective density of the carbon particles is lower than its physical density^54^. Assuming a lower density would result in even less particle mass, than already estimated with our calculation. The size distribution of the particles was also investigated with electron microscopy and the element analysis confirmed their chemical purity. Silicon and oxygen in the particle, measured by EDS might derive from siloxanes degassing from the silicone tube that connects the particle generator with the exposure station^55^.

16HBE14o- cell exposure to CNP leads to decreased TEER and increased FITC-Dextran permeability, indicating that CNP decrease barrier integrity in ALI-cultured 16HBE14o- cells (Fig. 2d, e). Diesel engine exhaust particles showed similar responses in previous studies despite differences in particle composition as well as cell culture and exposure conditions^30,31^.

In order to investigate our initial hypothesis that inhaled carbonaceous particles may have an impact on CFTR abundance, we tested the ability of CNP to alter *CFTR* gene expression. We detected a significant downregulation of CFTR expression at protein and transcript level following a 4-h exposure to CNP in the ALI-cultured 16HBE14o- cells. To our knowledge, we are the first group that reports such effects by CNP.

In our settings, such effect was not observed in cells exposed to the nitrogen-air mixture without particles (Supplement 4). This indicates that lower oxygen levels in the AES (10.9% versus 21%) per se, do not drive altered CFTR and oxidative stress responses in the 16HBE14o- cells, though some priming or additive effect on the observed CNP effects cannot be entirely ruled out. Downregulation of *CFTR* promotes epithelial-to-mesenchymal transition (EMT) in the development of breast cancer^56^. During EMT epithelial cells lose their polarized functionality and cell–cell contact^57^. CFTR is expressed on the apical surface of the cells^58^. Thus, losing cell polarity by EMT might be associated with decreased CFTR expression. EMT has also been associated with decreased expression of micro RNAs *MIR205* and *MIR126*^59,60^. Interestingly, *MIR126* was also reported to be downregulated in CF airway epithelial cells^61^. In our RNA-Seq analysis (Supplement 5, 6, 7) of CNP-exposed cells, *MIR205HG* expression was downregulated (Fig. 4b, Supplement 5) and while *MIR126* was not detected, the expression of one of its main targets *VEGFA* was highly increased (Fig. 4b, Supplement 5). Notably, reduced expression and function of CFTR has been previously associated with increased VEGF-A expression in airway epithelium^62^. Together with decreased barrier integrity and downregulated *CFTR* expression, these data might indicate first signs of EMT in CNP-exposed cells.

The molecular mechanisms that control CFTR downregulation at transcript and protein levels, upon CNP exposure, need further investigation.

At protein level, a possibility is that the exposure to carbon particles lead to CFTR internalization and degradation. Indeed, it was recently shown that cigarette smoke exposure induces a retrograde trafficking of CFTR to the endoplasmic reticulum in a clathrin/dynamin-dependent fashion^63^.

Furthermore, CNP-exposed cells exhibited increased expression of genes involved in the regulation of oxidative stress and it has been shown that oxidative stress leads to CFTR downregulation in T84 and Calu-3 cells^20^.

We showed a significant downregulation of *ANO1* and *TNFAIP3* in CNP-exposed cells. *ANO1* encodes a transmembrane channel that is critical for mucus secretion and production, it is thought to act in cooperation with CFTR and it can partially compensate for CFTR deficiency^64–66^. Reduced *TNFAIP3* and *CFTR* expression was reported in CF patients and seem to clinically correlate with lung function^67^. Moreover, *TNFAIP3* was downregulated in urban asthmatic patients^68^ indicating an important role of TNFAIP3 (A20) in airway disease. A20 is a negative regulator of NFκ-B activation exhibiting anti-inflammatory properties^69^. Decreased expression of *TNFAIP3* suggests therefore a pro-inflammatory response in CNP-exposed cells. However, this was not represented in our transcriptomic analysis, where most differentially regulated genes were downregulated, except for *GDF15*, which is reported to be a stress-induced cytokine^70^. In conclusion, downregulation of both, *ANO1* and *TNFAIP3* together with decreased *CFTR* expression in CNP-exposed 16HBE14o- cells suggest an unhealthy lung cells state.

Amongst other genes, *HMOX1* and *NFE2L2* were significantly increased, while *CAT* was significantly decreased. This expression pattern has been shown before in states of oxidative stress even upon exposure to cigarette smoke or airborne particles^71–73^. In this scenario, on the one hand, hydrogen peroxide accumulates in the cell due to decreased *CAT* expression. On the other hand, upregulated *HMOX1* will result in increased production of Fe^2+^ ions, which in turn support the Fenton reaction of hydrogen peroxide towards hydroxyl radicals. This induces a negative spiral to worsen oxidative stress in the cell and lead to DNA and cell damage and apoptosis^74,75^.

Our RNA-Seq analysis showed marked upregulation of several genes that are associated with an apoptotic state or signature of DNA damage. Apoptosis has been seen in CF^76–78^ and in bronchial epithelial cells exposed to carbon nanoparticles^51,79^. DNA damage and pathways involving the unfolded protein response and endoplasmic reticulum (ER) stress were also induced in CNP-exposed cells and seen in few CF patients^80,81^. Moreover, histone subunits were significantly downregulated, while *CDKN1A* was upregulated which has been described during DNA damage^82^.

Complementary to our current findings, ALI exposure studies with non-cystic fibrosis (CF) and CF airway epithelial cells have revealed marked further differences in susceptibility to nanoparticles. Using FITC-labelled model nanoparticles it was demonstrated that uptake and intercellular accumulation of these particles is enhanced in CF-cells in comparison to non-CF epithelial cells^83^. A role of CFTR in these uptake differences was confirmed via antisense oligonucleotide-based downregulation in NP-exposed epithelial cells. In another study, the effects of spark-generated CNP, as well as silver nanoparticles, were compared in CF versus normal bronchial epithelia at ALI-conditions. In the CF-cells enhanced necrosis as well as increased Caspase-3 activity and IL-6 release were observed^36^. Taken together our transcriptomic data indicate that CNP-exposed cells display an oxidative stress, DNA damage and apoptotic state. Thus, ALI-cultured 16HBE14o- cells are suitable for a toxicological assessment of spark-ablated nanoparticles after aerosol exposure.

## Conclusion

In conclusion, this study describes spark-ablated carbon nanoparticles in a realistic exposure of aerosols to decrease CFTR expression accompanied with transcriptomic signs of oxidative stress, apoptosis and DNA damage in ALI-cultured 16HBE14o- cells. Downregulation of *CFTR* might be a new mechanistic link to the aforementioned effects after CNP exposure. These effects, due to environmental factors, might explain the development of different phenotype severity in patients carrying the same CFTR mutation.

## Supplementary Information

Supplementary Information.

## Data Availability

The datasets generated and/or analysed during the current study are available at https://iufduesseldorf-my.sharepoint.com/:f:/g/personal/thach_nguyen_iuf-duesseldorf_de/EgVUZhXYif9KpwqNXWab_ZoBAEaKUDr_FI2g0OsaAa0Jdw?e=BTXlR8 or in the ArrayExpress repository (accession number E-MTAB-11389).

## Abbreviations

CFTR: Cystic fibrosis transmembrane conductance regulator
CNP: Carbon nanoparticles
16HBE14o−: Human bronchial epithelial cell line
ALI: Air–liquid interface
PM: Particulate matter
TRIC: Tricellulin
TEER: Transepithelial electrical resistance
FITC: Fluorescein-isothiocyanate
OCLN: Occludin
EM: Electron microscopy
ONT: Oxford nanopore
SMG: Ubmerged
E-cadherin: CDH1
KLF4: Krüppel-like factor 4
UBC: Ubiquitin C

## References

[1] Kreda SM, Davis CW, Rose MC (2012). CFTR, mucins, and mucus obstruction in cystic fibrosis. Cold Spring Harb. Perspect. Med..

[2] Collawn JF, Matalon S (2014). CFTR and lung homeostasis. Am. J. Physiol. Lung Cell. Mol. Physiol..

[3] Stahl M, Steinke E, Mall MA (2021). Quantification of phenotypic variability of lung disease in children with cystic fibrosis. Genes (Basel).

[4] Goeminne PC, Kicinski M, Vermeulen F, Fierens F, De Boeck K, Nemery B, Nawrot TS, Dupont LJ (2013). Impact of air pollution on cystic fibrosis pulmonary exacerbations: a case-crossover analysis. Chest.

[5] Psoter KJ, De Roos AJ, Wakefield J, Mayer JD, Rosenfeld M (2017). Air pollution exposure is associated with MRSA acquisition in young U.S. children with cystic fibrosis. BMC Pulm. Med..

[6] Donaldson K, Tran L, Jimenez LA, Duffin R, Newby DE, Mills N, MacNee W, Stone V (2005). Combustion-derived nanoparticles: a review of their toxicology following inhalation exposure. Part Fibre Toxicol..

[7] Li N, Sioutas C, Cho A, Schmitz D, Misra C, Sempf J, Wang M, Oberley T, Froines J, Nel A (2003). Ultrafine particulate pollutants induce oxidative stress and mitochondrial damage. Environ. Health Perspect..

[8] Stone V, Miller MR, Clift MJD, Elder A, Mills NL, Moller P, Schins RPF, Vogel U, Kreyling WG, Alstrup Jensen K (2017). Nanomaterials versus ambient ultrafine particles: an opportunity to exchange toxicology knowledge. Environ. Health Perspect..

[9] Tacu I, Kokalari I, Abollino O, Albrecht C, Malandrino M, Ferretti AM, Schins RPF, Fenoglio I (2021). Mechanistic insights into the role of iron, copper, and carbonaceous component on the oxidative potential of ultrafine particulate matter. Chem. Res. Toxicol..

[10] Ganguly K, Ettehadieh D, Upadhyay S, Takenaka S, Adler T, Karg E, Krombach F, Kreyling WG, Schulz H, Schmid O, Stoeger T (2017). Early pulmonary response is critical for extra-pulmonary carbon nanoparticle mediated effects: comparison of inhalation versus intra-arterial infusion exposures in mice. Part. Fibre Toxicol..

[11] Berger M, de Boer JD, Lutter R, Makkee M, Sterk PJ, Kemper EM, van der Zee JS (2017). Pulmonary challenge with carbon nanoparticles induces a dose-dependent increase in circulating leukocytes in healthy males. BMC Pulm. Med..

[12] Zhang R, Dai Y, Zhang X, Niu Y, Meng T, Li Y, Duan H, Bin P, Ye M, Jia X (2014). Reduced pulmonary function and increased pro-inflammatory cytokines in nanoscale carbon black-exposed workers. Part. Fibre Toxicol..

[13] Unfried K, Albrecht C, Klotz L-O, von Mikecz A, Grether-Beck S, Schins RPF (2007). Cellular responses to nanoparticles: Target structures and mechanisms. Nanotoxicology.

[14] Kim YM, Reed W, Lenz AG, Jaspers I, Silbajoris R, Nick HS, Samet JM (2005). Ultrafine carbon particles induce interleukin-8 gene transcription and p38 MAPK activation in normal human bronchial epithelial cells. Am. J. Physiol. Lung Cell. Mol. Physiol..

[15] Sydlik U, Gallitz I, Albrecht C, Abel J, Krutmann J, Unfried K (2009). The compatible solute ectoine protects against nanoparticle-induced neutrophilic lung inflammation. Am. J. Respir. Crit. Care Med..

[16] Weissenberg A, Sydlik U, Peuschel H, Schroeder P, Schneider M, Schins RP, Abel J, Unfried K (2010). Reactive oxygen species as mediators of membrane-dependent signaling induced by ultrafine particles. Free Radic. Biol. Med..

[17] Clunes LA, Davies CM, Coakley RD, Aleksandrov AA, Henderson AG, Zeman KL, Worthington EN, Gentzsch M, Kreda SM, Cholon D (2012). Cigarette smoke exposure induces CFTR internalization and insolubility, leading to airway surface liquid dehydration. FASEB J..

[18] Rab A, Rowe SM, Raju SV, Bebok Z, Matalon S, Collawn JF (2013). Cigarette smoke and CFTR: implications in the pathogenesis of COPD. Am. J. Physiol. Lung Cell. Mol. Physiol..

[19] Cantin AM, Hanrahan JW, Bilodeau G, Ellis L, Dupuis A, Liao J, Zielenski J, Durie P (2006). Cystic fibrosis transmembrane conductance regulator function is suppressed in cigarette smokers. Am. J Respir. Crit. Care Med..

[20] Cantin AM, Bilodeau G, Ouellet C, Liao J, Hanrahan JW (2006). Oxidant stress suppresses CFTR expression. Am. J. Physiol. Cell. Physiol..

[21] Callaghan, P. J., Ferrick, B., Rybakovsky, E., Thomas, S. & Mullin, J. M. Epithelial barrier function properties of the 16HBE14o- human bronchial epithelial cell culture model. *Biosci. Rep.* **40**, 10 (2020) 10.1042/BSR20201532 PMC7569203 32985670

[22] Forbes B, Shah A, Martin GP, Lansley AB (2003). The human bronchial epithelial cell line 16HBE14o- as a model system of the airways for studying drug transport. Int. J. Pharm..

[23] O'Farrell HE, Brown R, Brown Z, Milijevic B, Ristovski ZD, Bowman RV, Fong KM, Vaughan A, Yang IA (2021). E-cigarettes induce toxicity comparable to tobacco cigarettes in airway epithelium from patients with COPD. Toxicol. In Vitro.

[24] Haws C, Krouse ME, Xia Y, Gruenert DC, Wine JJ (1992). CFTR channels in immortalized human airway cells. Am. J. Physiol..

[25] Gruenert DC, Willems M, Cassiman JJ, Frizzell RA (2004). Established cell lines used in cystic fibrosis research. J. Cyst. Fibros.

[26] Clift MJ, Endes C, Vanhecke D, Wick P, Gehr P, Schins RP, Petri-Fink A, Rothen-Rutishauser B (2014). A comparative study of different in vitro lung cell culture systems to assess the most beneficial tool for screening the potential adverse effects of carbon nanotubes. Toxicol. Sci..

[27] Upadhyay S, Palmberg L (2018). Air-liquid interface: relevant in vitro models for investigating air pollutant-induced pulmonary toxicity. Toxicol. Sci..

[28] Frohlich E, Salar-Behzadi S (2014). Toxicological assessment of inhaled nanoparticles: role of in vivo, ex vivo, in vitro, and in silico studies. Int. J. Mol. Sci..

[29] Unfried K, Sydlik U, Bierhals K, Weissenberg A, Abel J (2008). Carbon nanoparticle-induced lung epithelial cell proliferation is mediated by receptor-dependent Akt activation. Am. J. Physiol. Lung Cell. Mol. Physiol..

[30] Smyth T, Veazey J, Eliseeva S, Chalupa D, Elder A, Georas SN (2020). Diesel exhaust particle exposure reduces expression of the epithelial tight junction protein Tricellulin. Part Fibre Toxicol..

[31] Lehmann AD, Blank F, Baum O, Gehr P, Rothen-Rutishauser BM (2009). Diesel exhaust particles modulate the tight junction protein occludin in lung cells in vitro. Part Fibre Toxicol..

[32] Zhou Z, Liu Y, Duan F, Qin M, Wu F, Sheng W, Yang L, Liu J, He K (2015). Transcriptomic analyses of the biological effects of airborne PM2.5 exposure on human bronchial epithelial cells. PLoS ONE.

[33] Loret T, Peyret E, Dubreuil M, Aguerre-Chariol O, Bressot C, le Bihan O, Amodeo T, Trouiller B, Braun A, Egles C, Lacroix G (2016). Air-liquid interface exposure to aerosols of poorly soluble nanomaterials induces different biological activation levels compared to exposure to suspensions. Part. Fibre Toxicol..

[34] Geiser, M., Jeannet, N., Fierz, M. & Burtscher, H. Evaluating Adverse Effects of Inhaled Nanoparticles by Realistic In Vitro Technology. *Nanomaterials (Basel)* **7**(2), 49 (2017). 10.3390/nano7020049 PMC5333034 28336883

[35] Mülhopt S, Dilger M, Diabaté S, Schlager C, Krebs T, Zimmermann R, Buters J, Oeder S, Wäscher T, Weiss C, Paur H-R (2016). Toxicity testing of combustion aerosols at the air–liquid interface with a self-contained and easy-to-use exposure system. J. Aerosol Sci..

[36] Jeannet N, Fierz M, Schneider S, Künzi L, Baumlin N, Salathe M, Burtscher H, Geiser M (2016). Acute toxicity of silver and carbon nanoaerosols to normal and cystic fibrosis human bronchial epithelial cells. Nanotoxicology.

[37] Brandenberger C, Rothen-Rutishauser B, Mühlfeld C, Schmid O, Ferron GA, Maier KL, Gehr P, Lenz AG (2010). Effects and uptake of gold nanoparticles deposited at the air-liquid interface of a human epithelial airway model. Toxicol. Appl. Pharmacol..

[38] Braakhuis, H. M. *et al.* An Air-liquid Interface Bronchial Epithelial Model for Realistic, Repeated Inhalation Exposure to Airborne Particles for Toxicity Testing. *J. Vis. Exp.* **13**, 59 (2020). 10.3791/61210 32478724

[39] He RW, Braakhuis HM, Vandebriel RJ, Staal YCM, Gremmer ER, Fokkens PHB, Kemp C, Vermeulen J, Westerink RHS, Cassee FR (2021). Optimization of an air-liquid interface in vitro cell co-culture model to estimate the hazard of aerosol exposures. J. Aerosol Sci..

[40] Mulhopt S, Schlager C, Berger M, Murugadoss S, Hoet PH, Krebs T, Paur HR, Stapf D (2020). A novel TEM grid sampler for airborne particles to measure the cell culture surface dose. Sci. Rep..

[41] Livak KJ, Schmittgen TD (2001). Analysis of relative gene expression data using real-time quantitative PCR and the 2(-Delta Delta C(T)) Method. Methods.

[42] Vogeley C, Sondermann NC, Woeste S, Momin AA, Gilardino V, Hartung F, Heinen M, Maass SK, Mescher M, Pollet M (2022). Unraveling the differential impact of PAHs and dioxin-like compounds on AKR1C3 reveals the EGFR extracellular domain as a critical determinant of the AHR response. Environ. Int..

[43] Lanfear R, Schalamun M, Kainer D, Wang W, Schwessinger B (2019). MinIONQC: fast and simple quality control for MinION sequencing data. Bioinformatics.

[44] Li H (2018). Minimap2: pairwise alignment for nucleotide sequences. Bioinformatics.

[45] Li H, Handsaker B, Wysoker A, Fennell T, Ruan J, Homer N, Marth G, Abecasis G, Durbin R (2009). Genome project data processing S: the sequence alignment/map format and SAMtools. Bioinformatics.

[46] Liao Y, Smyth GK, Shi W (2014). featureCounts: an efficient general purpose program for assigning sequence reads to genomic features. Bioinformatics.

[47] Anders S, Pyl PT, Huber W (2015). HTSeq–a Python framework to work with high-throughput sequencing data. Bioinformatics.

[48] Love MI, Huber W, Anders S (2014). Moderated estimation of fold change and dispersion for RNA-seq data with DESeq2. Genome Biol..

[49] Vogeley Christian, Nguyen Thach, Woeste Selina, Krutmann Jean, Haarmann-Stemmann Thomas, Rossi Andrea (2022). Rapid and simple analysis of short and long sequencing reads using DuesselporeTM. Frontiers in Genetics.

[50] Stöckmann D, Spannbrucker T, Ale-Agha N, Jakobs P, Goy C, Dyballa-Rukes N, Hornstein T, Kümper A, Kraegeloh A, Haendeler J, Unfried K (2018). Non-canonical activation of the epidermal growth factor receptor by carbon nanoparticles. Nanomaterials.

[51] Sydlik U, Bierhals K, Soufi M, Abel J, Schins RP, Unfried K (2006). Ultrafine carbon particles induce apoptosis and proliferation in rat lung epithelial cells via specific signaling pathways both using EGF-R. Am. J. Physiol. Lung Cell Mol. Physiol..

[52] Leikauf GD, Kim SH, Jang AS (2020). Mechanisms of ultrafine particle-induced respiratory health effects. Exp. Mol. Med..

[53] Geiser M, Stoeger T, Casaulta M, Chen S, Semmler-Behnke M, Bolle I, Takenaka S, Kreyling WG, Schulz H (2014). Biokinetics of nanoparticles and susceptibility to particulate exposure in a murine model of cystic fibrosis. Part Fibre Toxicol..

[54] Growney DJ, Fowler PW, Mykhaylyk OO, Fielding LA, Derry MJ, Aragrag N, Lamb GD, Armes SP (2015). Determination of effective particle density for sterically stabilized carbon black particles: effect of diblock copolymer stabilizer composition. Langmuir.

[55] Asbach C, Kaminski H, Lamboy Y, Schneiderwind U, Fierz M, Todea AM (2016). Silicone sampling tubes can cause drastic artifacts in measurements with aerosol instrumentation based on unipolar diffusion charging. Aerosol Sci. Technol..

[56] Zhang JT, Jiang XH, Xie C, Cheng H, Da Dong J, Wang Y, Fok KL, Zhang XH, Sun TT, Tsang LL (2013). Downregulation of CFTR promotes epithelial-to-mesenchymal transition and is associated with poor prognosis of breast cancer. Biochim. Biophys. Acta.

[57] Kalluri R, Weinberg RA (2009). The basics of epithelial-mesenchymal transition. J. Clin. Invest..

[58] Bertrand CA, Frizzell RA (2003). The role of regulated CFTR trafficking in epithelial secretion. Am. J. Physiol. Cell. Physiol..

[59] Gregory PA, Bert AG, Paterson EL, Barry SC, Tsykin A, Farshid G, Vadas MA, Khew-Goodall Y, Goodall GJ (2008). The miR-200 family and miR-205 regulate epithelial to mesenchymal transition by targeting ZEB1 and SIP1. Nat. Cell Biol..

[60] Jia Z, Zhang Y, Xu Q, Guo W, Guo A (2018). miR-126 suppresses epithelial-to-mesenchymal transition and metastasis by targeting PI3K/AKT/Snail signaling of lung cancer cells. Oncol. Lett..

[61] Oglesby IK, Bray IM, Chotirmall SH, Stallings RL, O'Neill SJ, McElvaney NG, Greene CM (2010). miR-126 is downregulated in cystic fibrosis airway epithelial cells and regulates TOM1 expression. J. Immunol..

[62] Martin C, Coolen N, Wu Y, Thevenot G, Touqui L, Pruliere-Escabasse V, Papon JF, Coste A, Escudier E, Dusser DJ (2013). CFTR dysfunction induces vascular endothelial growth factor synthesis in airway epithelium. Eur. Respir. J..

[63] Marklew AJ, Patel W, Moore PJ, Tan CD, Smith AJ, Sassano MF, Gray MA, Tarran R (2019). Cigarette smoke exposure induces retrograde trafficking of CFTR to the endoplasmic reticulum. Sci. Rep..

[64] Benedetto R, Ousingsawat J, Wanitchakool P, Zhang Y, Holtzman MJ, Amaral M, Rock JR, Schreiber R, Kunzelmann K (2017). Epithelial chloride transport by CFTR requires TMEM16A. Sci. Rep..

[65] Kunzelmann K, Ousingsawat J, Cabrita I, Dousova T, Bahr A, Janda M, Schreiber R, Benedetto R (2019). TMEM16A in cystic fibrosis: activating or inhibiting?. Front. Pharmacol..

[66] Ousingsawat J, Kongsuphol P, Schreiber R, Kunzelmann K (2011). CFTR and TMEM16A are separate but functionally related Cl- channels. Cell Physiol. Biochem..

[67] Kelly C, Williams MT, Elborn JS, Ennis M, Schock BC (2013). Expression of the inflammatory regulator A20 correlates with lung function in patients with cystic fibrosis. J. Cyst. Fibros..

[68] Krusche J, Twardziok M, Rehbach K, Bock A, Tsang MS, Schroder PC, Kumbrink J, Kirchner T, Xing Y, Riedler J (2019). TNF-alpha-induced protein 3 is a key player in childhood asthma development and environment-mediated protection. J. Allergy Clin. Immunol..

[69] Jarosz M, Olbert M, Wyszogrodzka G, Mlyniec K, Librowski T (2017). Antioxidant and anti-inflammatory effects of zinc. Zinc-dependent NF-kappaB signaling. Inflammopharmacology.

[70] Tavenier J, Rasmussen LJH, Andersen AL, Houlind MB, Langkilde A, Andersen O, Petersen J, Nehlin JO (2021). Association of GDF15 with inflammation and physical function during aging and recovery after acute hospitalization: a longitudinal study of older patients and age-matched controls. J. Gerontol. A Biol. Sci. Med. Sci..

[71] Chin BY, Trush MA, Choi AM, Risby TH (2003). Transcriptional regulation of the HO-1 gene in cultured macrophages exposed to model airborne particulate matter. Am. J. Physiol. Lung Cell. Mol. Physiol..

[72] Muller T, Gebel S (1994). Heme oxygenase expression in Swiss 3T3 cells following exposure to aqueous cigarette smoke fractions. Carcinogenesis.

[73] Quan X, Lim SO, Jung G (2011). Reactive oxygen species downregulate catalase expression via methylation of a CpG island in the Oct-1 promoter. FEBS Lett..

[74] Nandi A, Yan LJ, Jana CK, Das N (2019). Role of catalase in oxidative stress- and age-associated degenerative diseases. Oxid. Med. Cell. Longev..

[75] Redza-Dutordoir M, Averill-Bates DA (2016). Activation of apoptosis signalling pathways by reactive oxygen species. Biochim. Biophys. Acta.

[76] Soleti R, Porro C, Martinez MC (2013). Apoptotic process in cystic fibrosis cells. Apoptosis.

[77] Valdivieso AG, Santa-Coloma TA (2013). CFTR activity and mitochondrial function. Redox Biol..

[78] Chen Q, Pandi SPS, Kerrigan L, McElvaney NG, Greene CM, Elborn JS, Taggart CC, Weldon S (2018). Cystic fibrosis epithelial cells are primed for apoptosis as a result of increased Fas (CD95). J. Cyst. Fibros..

[79] Hussain S, Thomassen LC, Ferecatu I, Borot MC, Andreau K, Martens JA, Fleury J, Baeza-Squiban A, Marano F, Boland S (2010). Carbon black and titanium dioxide nanoparticles elicit distinct apoptotic pathways in bronchial epithelial cells. Part. Fibre Toxicol..

[80] Bartoszewski R, Rab A, Fu L, Bartoszewska S, Collawn J, Bebok Z (2011). CFTR expression regulation by the unfolded protein response. Methods Enzymol..

[81] Bartoszewski R, Rab A, Jurkuvenaite A, Mazur M, Wakefield J, Collawn JF, Bebok Z (2008). Activation of the unfolded protein response by deltaF508 CFTR. Am. J. Respir. Cell. Mol. Biol..

[82] Su C, Gao G, Schneider S, Helt C, Weiss C, O'Reilly MA, Bohmann D, Zhao J (2004). DNA damage induces downregulation of histone gene expression through the G1 checkpoint pathway. EMBO J..

[83] Ahmad Shama, Raemy David O., Loader Joan E., Kailey Jenai M, Neeves Keith B., White Carl W., Ahmad Aftab, Gehr Peter, Rothen-Rutishauser Barbara M. (2012). Interaction and Localization of Synthetic Nanoparticles in Healthy and Cystic Fibrosis Airway Epithelial Cells: Effect of Ozone Exposure. Journal of Aerosol Medicine and Pulmonary Drug Delivery.

