# Supplementary material for: Carbon nanoparticles adversely affect CFTR expression and toxicologically relevant pathways
Source: Sci Rep. 2022 Aug 22;12:14255. doi: 10.1038/s41598-022-18098-8 (PMC9395428; doi:10.1038/s41598-022-18098-8)
Supplement: Supplementary file 1 — Supplementary Information. [file 41598_2022_18098_MOESM1_ESM.docx]

**Supplement 1**

**Values for the physicochemical calculation of the carbon particle mass**

**Variables for calculation:**

Particle diameter: 44 nm

Particle number concentration: 6.36x10^6^/cm³

Spherical volume (nm³) = $\frac{\pi\cdot particle diameter^{3}}{6}$ = 44602.24

Spherical volume (cm³) = $\frac{spherical volume (nm^{3})}{{10}^{21}}$ = 4.46x10^-17^

Density of carbon: 2.26 g/cm³

Single particle mass (ng) = $(Spherical volume \left( \mathrm{cm}^{3} \right)\cdot density)\cdot{10}^{9}$ = 1.01x10^-7^

Mass concentration (ng/cm³) = $particle concentration \left( \frac{\mathrm{particle}}{\mathrm{cm}^{3}} \right)\cdot single particle mass (ng)$ = 6.41x10^-1^

Mass per exposure module (ng/min) = $mass concentration (\frac{ng}{cm^{3}})\cdot100$ = 64.1

Theoretically deposited mass (ng/min) = $mass per exposure module (\frac{ng}{\min})\cdot0.015$ = 0.962

Absolute particle mass on cells after exposure = $theoretically deposited mass (\frac{ng}{\min})\cdot240$ = **231**

**231 ng of carbon particles on the cells after a 4-hour exposure**

Considering an insert surface area of 4.67 cm², this corresponds to approx. **50 ng/cm²**

**Supplement 2**

**Oligonucleotide sequences for qPCR measurements**

| **Target** | **Forward sequence 5’-3’** | **Reverse sequence 5’-3’** |
| --- | --- | --- |
| *TJP1* | GCCAGCCTGCTAAACCTACT | GCCATCTCTTGCTGCCAAAC |
| *TJP2* | AAGCAGAGCGAACGAAGAGT | TTAGTTGCCAGACCCGTTCG |
| *CLDN1* | ATGTACGAGGGGCTGTGGAT | CAACGGTGGCCACAAAGATT |
| *OCLN* | CCAATGTCGAGGAGTGGGTT | TGCCATGGGACTGTCAACTC |
| *TRIC* | TCGTGATGCCCGACTATGTG | GGCTTTCCGAATGATGTGGC |
| *CDH1* | TTTCCACCAAAGTCACGCTG | GGCAGCTGATGGGAGGAATA |
| *CFTR* | CGTCATCAAAGCATGCCAACT | TTGCTCGTTGACCTCCACTC |
| *ANO1* | GACTACCACGAGGATGACAAGC | TCTCTGCACAGCACGTTCCA |
| *TNFAIP3* | CTGCCCAGGAATGCTACAGA | CAAATCTTCCCCGGTCTCTGT |
| *HMOX1* | ATGACACCAAGGACCAGAGCC | GTAAGGACCCATCGGAGAAGC |
| *HMOX2* | GGGAAAGGAGACATGCGTAA | CAAGAGTCCAGCAGCTAGGG |
| *NFE2L2* | ACATTGAGCAAGTTTGGGAG | TGTGGACTACAGTTACCTAC |
| *SOD2* | ATGTTGAGCCGGGCAGTG | GCGCGTTGATGTGAGGTTCC |
| *SOD1* | GGCCGATGTGTCTATTGAAGA | GGGCCTCAGACTACATCCAA |
| *CAT* | CAAACCGCACGCTATGGCT | GCTCCAGTGGTCAGGACATC |
| *GPX1* | GCATCAGGAGAACGCCAAGA | TCGAAGAGCATGAAGTTGGGC |
| *PGK1* | TTGATGAAGAAGGAGCTGAACT | GTCTGCAACTTTAGCTCCGC |

**Supplement 3**

**Characterization of barrier integrity in 16HBE14o- cells under ALI conditions**


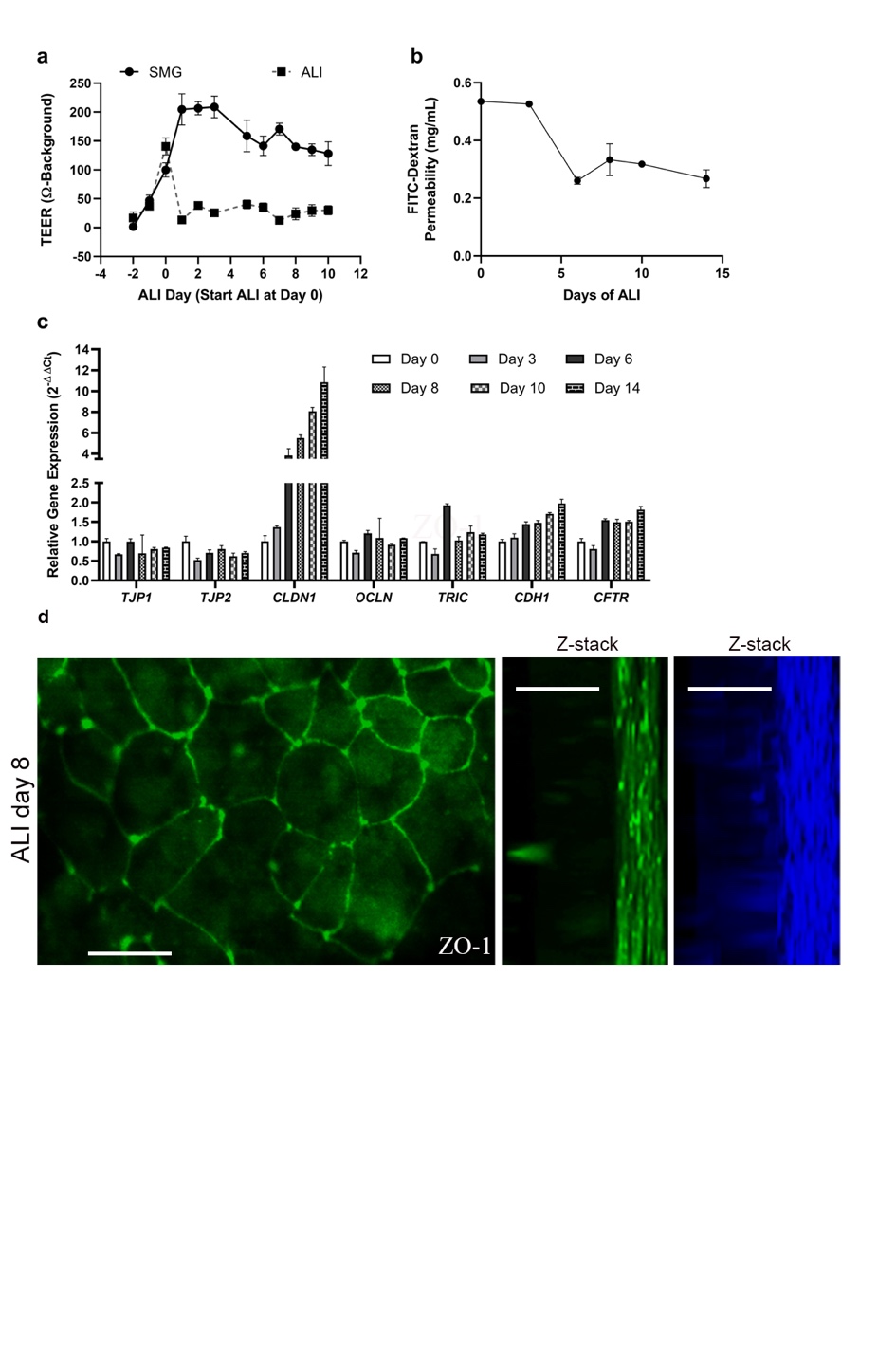


**Supplement 3.** Characterization of barrier integrity in 16HBE14o- cells under ALI conditions. **a** For TEER development, cells were seeded in 6 well Transwell inserts with 6x10^5^ cells per insert and cultured under submerged (SMG) conditions for 4 days before changing to ALI conditions for a part of the inserts. Cells were cultured for 10 more days either at ALI or under SMG conditions and TEER was recorded from day -2 every day except for day 4. Data are mean ± SD of three Transwell inserts as technical replicates. Each Transwell insert was measured at three cavities and the mean used for calculation. **b** FITC-Dextran permeability was measured as described in the method section between day 0 and day 14 of ALI-cultured 16HBE14o- cells. Data are mean ± SD of one (day 3, day 10) or two (day 0, day 6, day 8, day 14) Transwell replicates. Each Transwell was measured in triplicates. **c** During 14 days of ALI-cultured 16HBE140- cells RNA was isolated at different days and gene expression for tight junction markers, *CDH1* and *CFTR* was measured by qPCR and normalized to *PGK1* expression. Data are mean ± SD of one experiment with two technical replicates. d. ZO-1 and DAPI staining in 16HBE140- ALI at 8 days, Z-stack on the left (scale bar 10 um).

**Supplement 4**

**
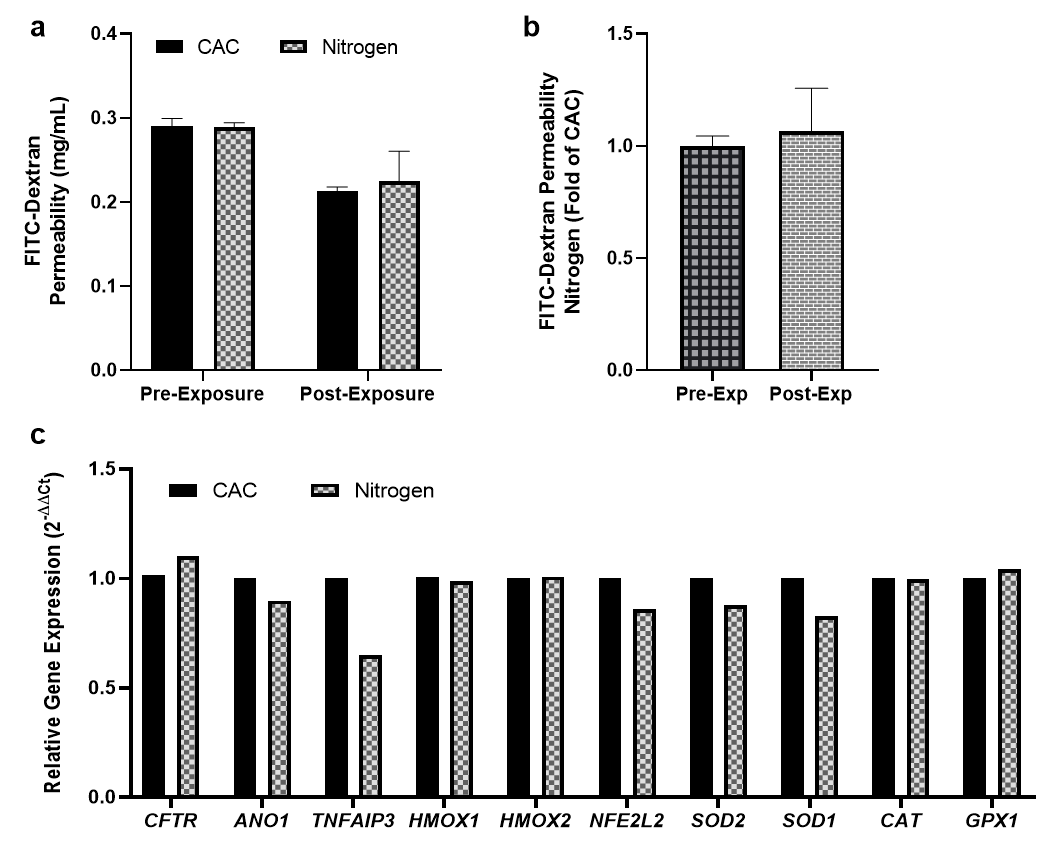
**

**Supplement 4.** Effect of nitrogen carrier gas supply on FITC-Dextran permeability and gene expression in ALI-cultured 16HBE cells. ALI-cultured cells were exposed to nitrogen-air mixture generated with nitrogen at 8 L/min gas flow or clean air at day 5 of ALI. **a** FITC-Dextran permeability pre- and post-exposure to nitrogen or clean air control (CAC) displayed as mg/mL. **b** FITC-Dextran permeability in nitrogen-exposed cells pre- and post-exposure displayed as fold of CAC. For **a** and **b**: Data are mean ± SD of a single experiment with three inserts as technical replicates. **c** Expression of *CFTR*, *ANO1*, *TNFAIP3* and markers of oxidative stress in nitrogen-exposed 16HBE14o- cells compared to CAC. Data are normalized to *PGK1* expression. Data are mean of a single experiment measured with two technical replicates

**Supplement 5**

**Expression of different genes analyzed after RNA sequencing from CNP-exposed 16HBE14o- cells.**

**
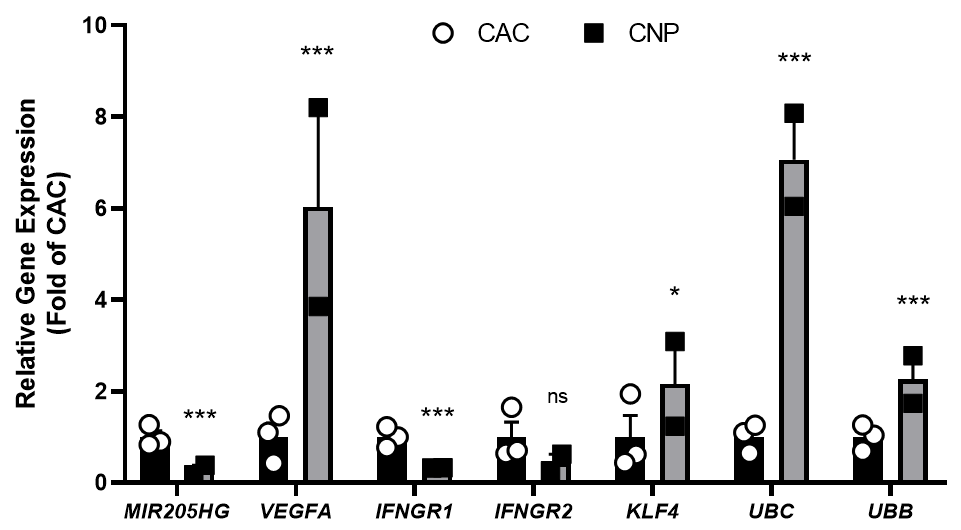
**

**Supplement 5.** Expression of different genes analyzed after RNA sequencing from CNP-exposed 16HBE14o- cells. Isolated RNA was subjected to Long Read Nanopore Sequencing to analyze expression of different genes involved in various described mechanisms of *CFTR* downregulation. Data are mean ± SEM of two (CNP) or three (CAC) independent experiments. Statistical significance was calculated using the DESeq2 algorithm as described in the method section. ns = not significant; * p < 0.05; *** p < 0.001

**Supplement 6**

**
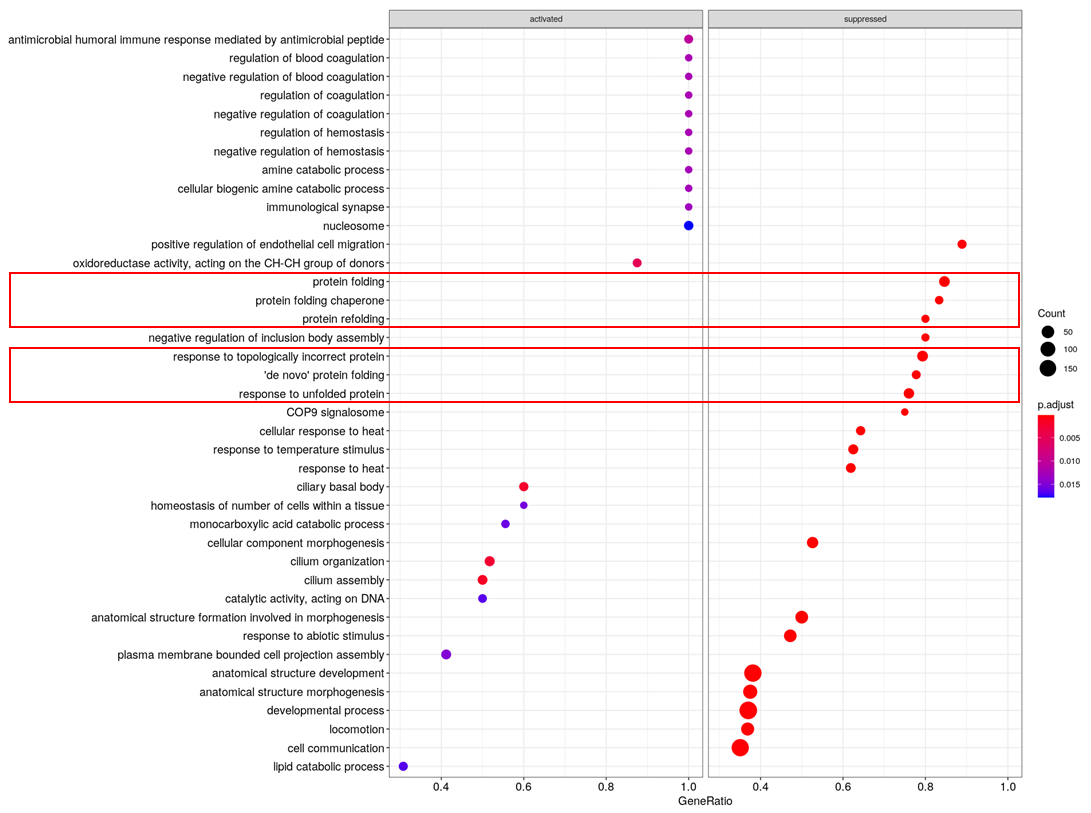
**

**Supplement 6.** Dot Plot Pathway analysis of RNA sequencing data

**Supplement 7**


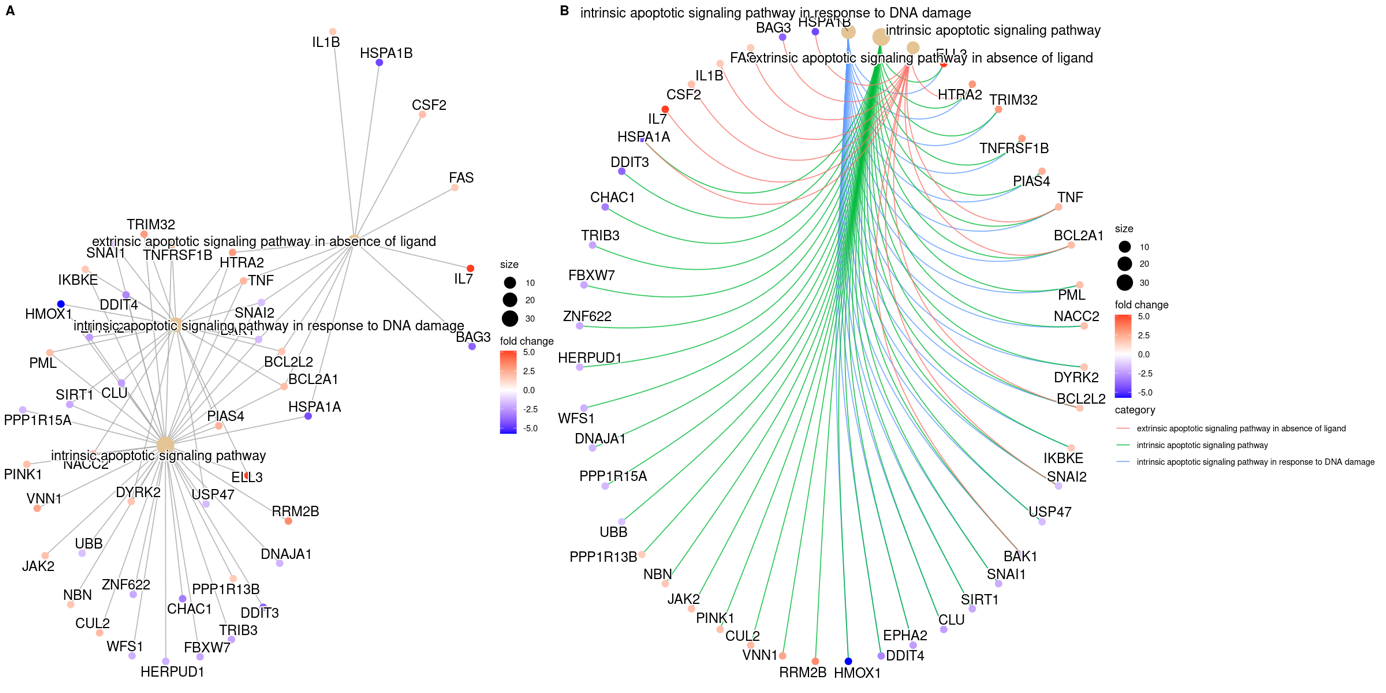
**Supplement 7**. Apoptosis Pathway analysis of RNA sequencing data

**Supplement 8**


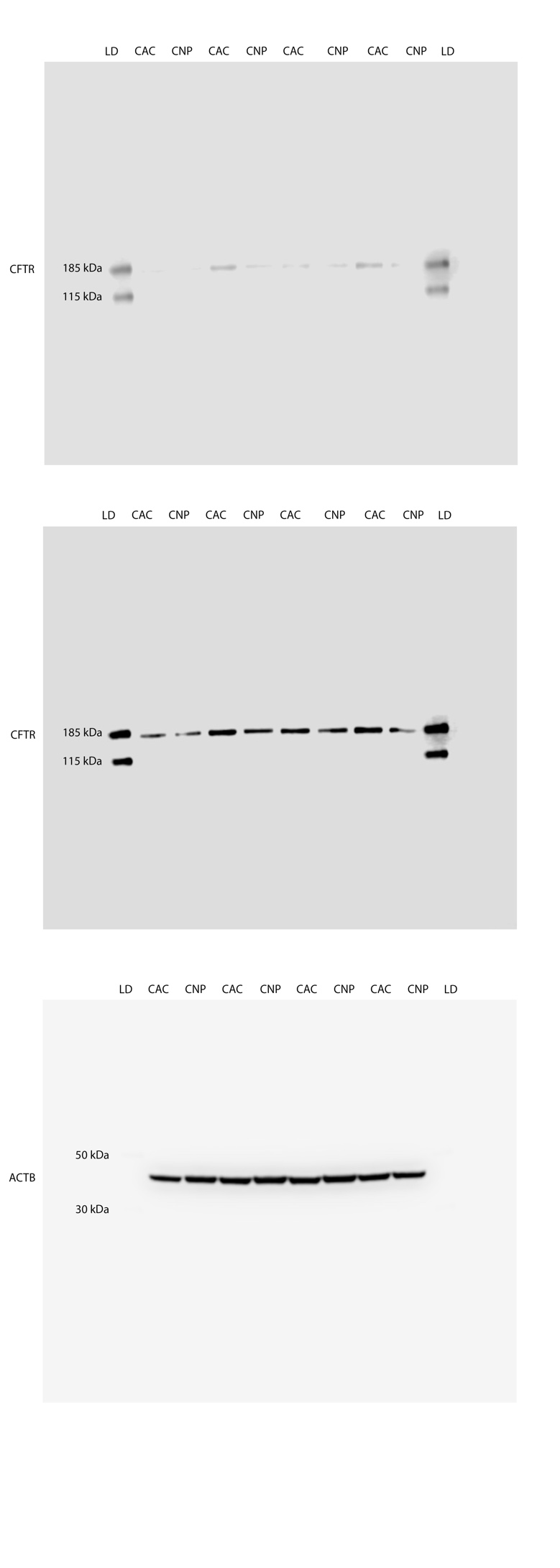


**Supplement 8.** Western Blot analysis of CFTR (top) at low and high exposure and ACTB (bottom) on 16HBE14o- not exposed (CAC) or CNP-exposed from lane 2 to 9 representing different biological replicates.

LD represents the ladder lane.
